# Combined long nerve allograft and nerve transfer for functional recovery of ulnar nerve: case study with longitudinal clinical and neurodiagnostic follow-up

**DOI:** 10.3389/fnins.2025.1666023

**Published:** 2025-09-17

**Authors:** Caroline Cushman, Naveen Arunachalam Sakthiyendran, Evan J. Hernandez, Robert N. Kurtzke, Brendan J. MacKay

**Affiliations:** ^1^Department of Orthopedic Hand Surgery, Texas Tech University Health Sciences Center, Lubbock, TX, United States; ^2^Department of Neurosurgery, Boston University Chobanian and Avedisian School of Medicine, Boston, MA, United States; ^3^Neurology Center of Fairfax, Fairfax, VA, United States

**Keywords:** allograft, nerve transfer, peripheral nerve surgery, reconstruction, microsurgery

## Abstract

**Background:**

Peripheral nerve injuries involving large gaps (>50 mm) are associated with poor outcomes due to delayed axonal regeneration and limited reconstructive options. While autografts are traditionally the gold standard, their use is limited by donor site morbidity and length constraints. Processed nerve allografts have emerged as an alternative, but data on their long-term efficacy, particularly for gaps ≥60 mm remain limited.

**Case report:**

We present a case of a 15-year-old male with a 68-mm ulnar nerve gap following trauma reconstructed acutely with a processed nerve allograft. This procedure was performed in conjunction with an anterior interosseous nerve (AIN) to ulnar motor branch transfer and ulnar nerve decompression of potential entrapment sites.

**Results:**

Serial assessments over 4.5 years demonstrated substantial recovery. By 16 months, the patient had regained strong grip strength, full range of motion, and near-normal sensory thresholds. At final follow-up, he had returned to all activities without limitations. Serial EMGs confirmed early nascent motor unit recruitment by 3 months, progressive reinnervation at 16 months, and persistent low-amplitude responses at 54 months, suggestive of ongoing but incomplete reinnervation.

**Conclusion:**

This case provides the longest known electrodiagnostic follow-up of a long-gap ulnar nerve allograft reconstruction. It supports the feasibility of processed allografts for gaps ≤70 mm and emphasizes the value of long-term EMG monitoring in tracking regeneration. These findings contribute critical data to a sparsely studied domain and help define expectations for complex nerve repairs.

## 1 Introduction

Peripheral nerve injuries (PNIs) occur in approximately 2%–3% of trauma patients, often resulting in severe motor and sensory deficits and long-term disability. Large nerve-gap injuries – particularly gaps exceeding 50 mm – are especially challenging: regeneration must span an extended distance, during which denervated Schwann cells and end-organs/muscles may atrophy (Foy et al., 2021). Following injury, the distal nerve segment undergoes Wallerian degeneration, characterized by axonal breakdown, demyelination, and clearance of cellular debris, which prepares the pathway for regeneration but also sets a limited time window for effective repair (Foy et al., 2021). Hence, in general, outcomes decline more rapidly as gap length, patient age, or delay to repair increase (Safa et al., 2020; Foy et al., 2021).

Although autologous nerve grafting has historically been the gold-standard for reconstituting peripheral nerve continuity, it requires harvest from a donor nerve with attendant morbidity (sensory loss, wound complications) and is limited in its ability to cover longer injury gaps. Studies have shown that longer autografts incur increased Schwann cell senescence and diminished regeneration (Safa et al., 2020). Consequently, there is growing interest in processed acellular nerve allografts for long-gap repairs, as they avoid donor-site morbidity and provide ready scaffolds without requiring removal of tissue (Safa et al., 2020). Registry data suggest that processed allografts yield high rates of “meaningful recovery” even in mixed motor-sensory nerves up to 70 mm long (Cho et al., 2012; Safa et al., 2020). Despite these promising results, there remains relatively little published data on outcomes of very long-gap (≥60 mm) peripheral nerve repairs, especially with long-term follow-up. While other cases similar to our patient’s exist in the literature, these cases involved substantial delays, older patients, and specialized adjuncts (e.g., PRP), making comparisons to immediate repair with processed allograft difficult.

In this case report and review of the literature, we detail a 4.5-year follow-up of a teenager with a 68 mm proximal ulnar nerve gap reconstructed sub-acutely with processed allograft followed up an anterior interosseous-to-ulnar nerve transfer (AIN-ulnar). Moreover, we present serial electromyography and quantitative sensory/motor testing to highlight the extent of recovery. This case is unique in its long gap length, combined nerve transfer, and comprehensive long-term objective monitoring, and it adds critical insight on the utility of nerve allografts for large and involved peripheral nerve defects.

## 2 Case presentation

A 15-year-old male was involved in a motor vehicle collision and sustained a complex left upper arm laceration along with a left femur fracture. He underwent urgent femur fixation and debridement of the brachial plexus region. At 1-month post-injury, he reported complete numbness of the left pinky finger, near-complete loss of sensation in the ring finger, and severe weakness/clawing of the ring and pinky finger intrinsic muscles. Examination revealed left ulnar nerve palsy (no intrinsic hand motion, positive Wartenberg’s sign, escape sign) and hypoesthesia in the ulnar nerve distribution. The median and radial nerves appeared intact. Given the clear clinical picture, the patient proceeded to operative exploration without further imaging.

### 2.1 First surgery at 1-month post-injury

The prior laceration was extended to expose the neurovascular bundle. Intraoperatively, the ulnar nerve was found to be completely transected just proximal to the cubital tunnel, leaving a 68 mm gap after debridement of neuroma. The surgeon excised residual scar and prepared healthy proximal and distal nerve stumps. A 70 mm processed nerve allograft (matched to ∼4–5 mm diameter) was trimmed to 68 mm and sutured end-to-end between the ulnar nerve stumps using 7-0 prolene. Small porcine submucosa protectors were placed proximally and distally, and fibrin glue was applied to reinforce the coaptation. The ulnar nerve was transposed anteriorly at the elbow to prevent tethering and shorten the nerve gap, and proximal and distal decompressions were performed (release of cubital tunnel and Guyon’s canal) to optimize regeneration conditions. Finally, the left anterior interosseous nerve (AIN) branch to the pronator quadratus – which was identified distal to its musculocutaneous branches – was identified and coapted in an end-to-side fashion to the motor fascicles of the proximal ulnar nerve branch supplying the intrinsic hand muscles. The incisions were irrigated and closed. The patient’s postoperative course was uncomplicated.

On post-operative day 8, the patient reported decreased neuropathic pain along the ring finger. He continued to have numbness in the small finger. Physical exam showed intrinsic muscle paralysis and positive Tinel’s at the repair site and cubital tunnel, but he tolerated gentle range-of-motion therapy for the elbow and hand.

### 2.2 Second surgery at 7 months post-initial repair

**FIGURE 1:**
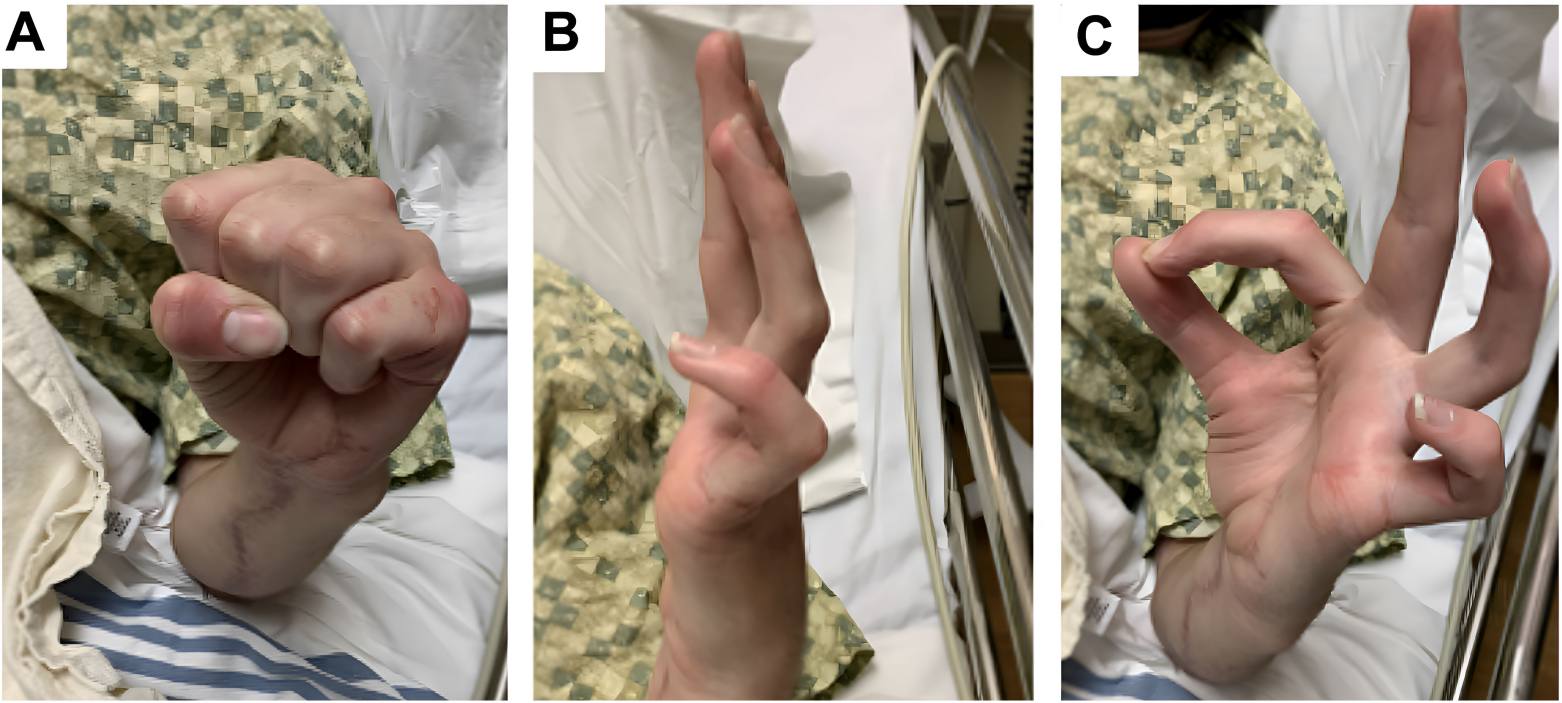
Clinical photographs taken 7 months after initial nerve reconstruction, prior to tendon transfer. **(A)** Image shows the hand at rest showing persistent clawing deformity. **(B**) Image shows patient attempting active finger extension but cannot fully extend the PIP/DIP joints, especially in the ulnar digits (ring and little finger). MCPs remain relatively extended representing incomplete extension due to intrinsic muscle weakness. **(C)** Image shows patient attempting opposition of the thumb and index digits – thumb IP joint is flexed, and the index finger DIP joint is flexed, suggesting donor-site weakness related to sacrifice of the anterior interosseous nerve branch to the pronator quadratus, rather than a generalized median nerve deficit.

To stabilize the persistent hyperextension of the MCP joints at the 4th–5th digits, the patient underwent planned secondary procedures. These included volar plate advancement at the 4th and 5th metacarpophalangeal joints (to correct finger flexion contractures), and a tendon transfer: the left abductor pollicis longus tendon was rerouted to the first dorsal interosseous (FDI) muscle to augment ulnar intrinsic function. No further nerve repair was performed at this stage. Postoperatively the hand was mobilized; by 9 months post–initial repair (roughly 2 months after the tendon transfer) the patient had 100% active range of motion of the wrist and all fingers and could make a composite fist (Figure 1). The small finger remained the least sensate area.

### 2.3 Long term functional follow-up

**FIGURE 2:**
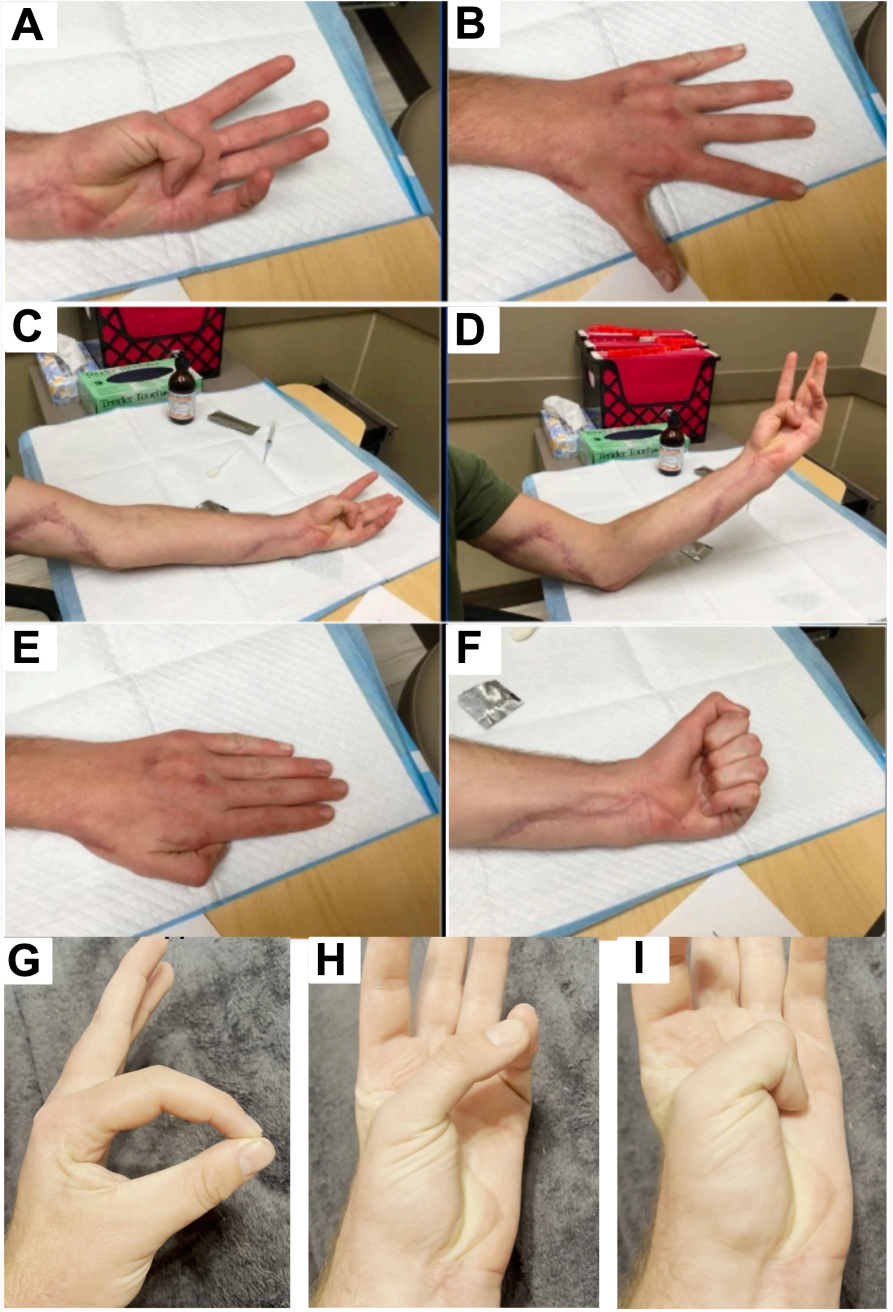
Clinical images obtained 7 months after tendon transfer and 14 months after initial nerve repair showing improved hand posture, full finger extension, and resolution of clawing. **(A)** Image shows patient flexing the thumb and achieving gross complete range of motion, indicating recovery of the flexor pollicis longus. **(B)** Image shows full finger extension with an open hand. MCP, PIP, and DIP joints are all fully extended without residual clawing deformity. **(C)** Image shows flexion/opposition of the thumb into the palm of the hand in a relaxed elbow extension position, showing restored median nerve function (via anterior interosseous nerve) allowing flexion of the thumb IP joint. The well-healing surgical scar from the procedures along the volar forearm can also be visualized. **(D)** Image shows flexion/opposition of the thumb into the palm of the hand in an elbow flexion, arm-elevation position. **(E)** Image shows hand at rest in a dorsal view with fingers extended and relaxed. There is no evidence of clawing or abnormal posturing, indicating restored resting hand posture. **(F)** Image shows patient demonstrating a full fist grip, indicating ability to fully flex the fingers, and restored flexor function across the MCP, PIP, and DIP joints. **(G)** Opposition of thumb and index finger confirms preserved flexor pollicis longus function. **(H)** Thumb palmar abduction with full extension demonstrates maintained carpometacarpal and MCP mobility. **(I)** Thumb opposition into the palm demonstrates continued anterior interosseous–mediated flexion at the IP joint, confirming long-term durability of motor recovery.

The patient was evaluated serially with motor and sensory testing and electrodiagnostic assessment. By 12–16 months after the initial surgery, substantial functional gains were observed (Figure 2). At 14.5 months, left grip strength was 35 kg (versus 64 kg on the right), key pinch 4.5 kg (right 15 kg), tip pinch 2.0 kg (right 5.0 kg), and 3-jaw chuck pinch 1.0 kg (right 7.0 kg). Active range of motion was full. On Semmes-Weinstein monofilament testing, all digits except the small finger had returned to light-touch normal thresholds (2.83–3.61 g); the small finger palmar tip still required protective pressure (3.61–4.31 g). Pain was minimal (VAS 3), and his QuickDASH disability score was 15.9% (near normal). By 16 months, grip strength increased to 50 kg, 3-jaw pinch to 5 kg, and sensory thresholds had normalized across all ulnar-innervated sites (Semmes 2.83 g), with QuickDASH improving to 11.4%. These results reflected functional independence in daily activities (Table 1). At 33 months post-repair, the patient maintained full range of motion with a VAS pain score of 0. Semmes-Weinstein testing revealed thresholds of 4.31 in the lateral dorsal fourth digit, 3.61 in the ulnar volar digit, and 6.65 in the lateral hand (Table 1). A video of the patient’s active range of motion. Of finger movement was also recorded at 47 months post-repair (Supplementary Video 1).

**TABLE 1:** Serial quantitative assessments of sensory and motor recovery following ulnar nerve allograft repair.

Visit	Semmes-weinstein	VAS score	Grip (lbs)	Key (lbs)	Tip (lbs)	3 Jaw (lbs)	Tinel’s sign	Total active motion
Follow-up 1 (2 months post allograft)	Lateral 4th: 2.83/Dorsal 5th: 6.65/Volar 5th: 4.56/Lateral hand: 2.83	1	R:60 L:25	R:10 L:3	R:7 L:3	R:6 L:2.5	–	–
Follow-up 2 (4 months post allograft)	Lateral 4th: 6.65/5th: No sensation/Lateral hand: No sensation	0	R:68 L:23	R:9 L:1.5	-	-	At target site	Clawing to 4^th^ and 5^th^ fingers
**Tendon transfer (7 months post allograft)**								
Follow-up 3 (9 months post allograft)	Lateral volar 4th: 4.56/Volar 5th: 6.65/Lateral volar hand: 4.56	–	L:25	L:2	L:2	L:3	At wrist crease	100%
Follow-up 4 (14 months post allograft)	Lateral 4th: 2.83/Dorsal 5th: 3.61/Volar 5th: 4.31/Lateral dorsal hand: 2.83/Lateral volar hand: 3.61	3	R:64 L:35	R:15 L:4.5	R:5 L:2	R:7 L:1	Not observed	100%
Follow-up 5 (16 months post allograft)	All normal	0	R:50 L:74	R:14 L:4	R:4 L:1.5	R:11 L:1.5	At Carpal tunnel	100%
Follow-up 6 (19 months post allograft)	5th: Pressure only/Lateral hand: 3.61/All others normal	0	R:50 L:74	R:4	R:2	R:4	At 1 cm proximal to wrist crease	100%
Follow-up 7 (33 months post allograft)	Lateral dorsal 4th: 4.31/Lateral volar 4th: 3.61/Lateral hand: 6.65	0	–	–	–	–	–	100%

Data include Semmes-Weinstein monofilament thresholds, visual analog scale (VAS) pain scores, grip and pinch strength (key, tip, and 3-jaw chuck), presence and location of Tinel’s sign, and total active motion (TAM) of the affected hand across seven follow-up visits from 2 to 33 months postoperatively. Note the progressive improvement in strength and sensation, resolution of clawing. following tendon transfer, and sustained 100% TAM, throughout follow-up; R, right (unaffected); L, left (affected); VAS, visual analog scale; lbs, pounds.

### 2.4 Neurophysiology and nerve conduction study data

Needle EMG and nerve conduction studies were performed at 3, 16, and 54 months. At 3 months, there were early motor unit potentials in ulnar-innervated muscles and absent sensory responses, consistent with nascent reinnervation. By 16 months, the ulnar motor nerve showed low-amplitude CMAPs to the ring-finger intrinsic muscles, and motor units were recruited on volitional testing, though amplitudes were reduced and latencies prolonged. Sensory nerve action potentials (ulnar sensory) had returned but remained low. By 4.5 years, conduction velocities showed minimal improvement, but motor CMAP amplitudes were still subnormal, and the waveform showed dispersion, suggesting incomplete reinnervation or a focal conduction delay (possibly residual entrapment at the elbow, despite initial transposition). Nonetheless, EMG demonstrated ongoing motor unit recruitment in the ulnar-innervated hand muscles at 4.5 years, confirming sustained regeneration (Table 2).

**TABLE 2:** Electromyography (EMG) and nerve conduction study (NCS) data at 3-, 16- and 54-month follow-up

EMG timeline	Nerve	Distal peak sensory latency (ms)	Sensory amp. (uV)	Distal motor latency (ms)	Motor amp. (mV)	Conduction velocity (m/s)	F waves (ms)
EMG at 3 months post-initial nerve grafting and transfer	L. Median	3.2	51	4.2	7.8	62	–
	L. Ulnar	0	0	4.7	0.2	37	–
	L. Radial	2.6	31	1.9	6.0	60	–
EMG at 16 months post-initial nerve grafting and transfer	L. Median	2.3	35	3.4	20	59	–
	L. Ulnar	1.9	30	2.7	19	65	26.5
	L. Radial	–	–	–	–	–	*
EMG at 54 months post-initial nerve grafting and transfer	L. Median	1.7	100	3.6	10	61	27–28
	L. Ulnar	1.8	10	4.3	3	53	34–36
	L. Radial	2.9	30	2.2	5	59	24–25

First electromyography (EMG) and nerve conduction study (NCS) at 3 months post-repair demonstrated early motor unit potentials in ulnar-innervated muscles with absent sensory responses, indicating nascent reinnervation. At 16 months, repeat studies showed recruitment of ulnar motor units with low-amplitude CMAPs and re-emergence of SNAPs, reflecting ongoing regeneration. By 4.5 years, EMG revealed persistently low-amplitude CMAPs with waveform dispersion across the elbow, suggestive of incomplete reinnervation and possible focal conduction delay consistent with residual ulnar nerve entrapment.

* F-waves for the radial nerve at 16 months were not recorded. ms, millisecond; *uV*, microvolts; Amp, Amplitude; m/s, meter/second.

Throughout follow-up, the patient reported only minimal symptoms. No wound complications or immune reactions to the grafts occurred. At follow-up 4.5 years post-initial repair, the patient had strong hand motor function (able to make a fist and perform fine pinch tasks) and normal ulnar nerve sensation by formal testing (Table 1). From a functional perspective, the patient returned to all pre-injury activities without restrictions.

## 3 Discussion

Peripheral nerve injuries involving long gaps (>50 mm) present substantial challenges to functional recovery due to delayed axonal regeneration, reduced Schwann cell migration, and the absence of a universally accepted gold standard for reconstruction (Höke, 2006; Carvalho et al., 2019). Autologous nerve grafting remains the historical standard; however, its utility diminishes in large defects due to the need for donor nerve sacrifice, length limitations, and technical challenges such as cable grafting, which may reduce axon density (Moore et al., 2011; Tang et al., 2013; Salomon et al., 2016; Draeger et al., 2017; Contreras et al., 2022). Processed nerve allografts have emerged as a promising alternative, offering the benefit of off-the-shelf availability and eliminating donor site morbidity (Tang et al., 2013; Salomon et al., 2016). These grafts preserve the endoneurial scaffold, are enzymatically prepared to promote regeneration, and have been FDA-approved for gaps up to 70 mm (Moore et al., 2011). Despite increasing clinical use, data on long-term outcomes, especially those confirmed by electrodiagnostic testing, remain scarce. Of those reported, a multicenter series reported 82% of allograft repairs (sensory, mixed, or motor nerves) achieving functional recovery for grafts up to 70 mm^2^, and RANGER registry data demonstrated S3/M4 (S3: return of protective sensation, including pain and tactile discrimination; M4: active movement against resistance) or better recovery in 86% of cases (up to 50 mm gaps) including 69% success rate for patient who received ulnar nerve repairs (Safa et al., 2020).

This case of a 68-mm ulnar nerve injury reconstructed with decellularized nerve allograft contributes meaningfully to this limited body of evidence. We present a 5-year clinical and electrodiagnostic follow-up, which, to our knowledge, is the longest reported to date for a long nerve allograft. While many studies rely on subjective or qualitative metrics such as grip strength, range of motion, and patient-reported outcomes, this case demonstrates how serial electromyography (EMG) can provide an objective, physiologically grounded assessment of axonal regeneration, conduction velocity, and evolving nerve function (Raez et al., 2006).

Only a few studies in the literature have similarly attempted to characterize long-gap repairs using EMG, though none provide follow-up of comparable duration. Foy et al. (2021) described a 56-year-old patient who underwent autograft-based repair of a 9-cm ulnar nerve gap using sural nerve segments in combination with platelet-rich plasma (PRP) and a collagen tube. EMG at 1.75 years postoperatively revealed prolonged latency and reduced amplitude, suggesting partial motor reinnervation with poor muscle fiber recruitment (Foy et al., 2021). However, sensory recovery was predominant, with EMG demonstrating topographically correct sensitivity across 11-cm gaps and full restoration of sensation (Foy et al., 2021). This case accentuates the common finding that sensory axon recovery tends to outpace motor recovery in large-gap injuries. In a second study, Kuffler et al. (2011) followed a 48-year-old patient who underwent ulnar nerve repair 3.25 years after trauma to address a 12-cm gap. Over 2 years, EMG showed gradual increases in conduction velocity and emerging motor function, including the generation of 1 kg of force in the ring and small fingers (Kuffler et al., 2011). However, sensory amplitude remained low, mirroring the findings from Foy et al. (2021) (Kuffler et al., 2011). The patient achieved functional improvement sufficient to return to light-duty work. EMG was key in documenting this incremental recovery.

Compared to these reports, our patient, 15 years old at the time of injury, demonstrated earlier and more consistent motor recovery, likely reflecting greater neuroplasticity and regenerative potential. The favorable outcome in this case is likely influenced by the patient’s young age, which confers faster axonal regeneration and greater cortical plasticity compared to adults. This neurobiological advantage may partly explain the superior recovery compared to prior reports in older patients with comorbidities. EMG at 3 months revealed low-amplitude responses in the intrinsic hand muscles, indicative of early axonal continuity. At 16 months, clinical grip strength in the affected hand equaled the contralateral side, and EMG demonstrated sustained reinnervation. However, despite these early gains, the 4.5-year EMG showed persistently low motor amplitudes and signs of ulnar nerve entrapment at the elbow, suggesting late-stage conduction impairment. This pattern illustrates the dynamic nature of nerve recovery, where early reinnervation may plateau or regress due to secondary factors such as fibrosis or entrapment. Additionally, the unusually high motor amplitude at 16 months may also reflect technical variability, electrode placement, or volume conduction from the reinnervated median nerve, while the subsequent decline at 54 months may represent late conduction block, entrapment, or physiologic pruning of reinnervated motor units. These fluctuations highlight the limitations of single-timepoint EMG interpretation and underscore the value of serial review.

These findings support the critical role of EMG in evaluating long-term outcomes following nerve repair. While grip strength, range of motion, and patient-reported measures are indispensable for functional assessment, they lack the specificity to detect subclinical conduction abnormalities or differentiate true axonal regeneration from compensatory reinnervation. EMG offers an objective window into the electrophysiological status of the nerve, enabling identification of incomplete regeneration, conduction block, or secondary complications such as entrapment, factors that may go unnoticed through physical examination alone.

Our case emphasizes the need for more standardized, longitudinal outcome measures in peripheral nerve surgery. Neither the Foy et al nor Kuffler et al. (2011) studies reported follow-up beyond two years, nor did they explore late-stage electrophysiological complications (Foy et al., 2021). The integration of serial EMG, combined with functional testing and validated patient-reported outcome tools, would provide a more comprehensive and reproducible framework for evaluating the success of large-gap repairs. Moreover, while our patient represents an ideal recovery scenario, young, healthy, and without comorbidities, outcomes in older or more complex patients may differ significantly. The combined reconstruction also complicates attribution of recovery to either the allograft or the nerve transfer. The return of thumb IP joint flexion (median/AIN function) can be attributed to the AIN donor branch itself, while recovery of intrinsic hand strength and claw correction likely reflects ulnar axonal regeneration through the allograft. Thus, the AIN transfer served as an early protective reinnervation, whereas the allograft supported long-term ulnar recovery.

Despite the novelty of our case, this study and report is not without limitations. Given the complexity of this patient’s nerve reconstruction and the involvement of multiple neurologists performing EMG studies at different time points, there was some variability in the electrodiagnostic assessments. To mitigate this inconsistency, a third-party neurologist with fellowship training, board certification, and specific expertise in major nerve reconstruction EMG was enlisted to independently review all three EMGs alongside detailed clinical histories and relevant imaging. Upon careful review, discrepancies emerged, particularly concerning measurements of the left median and ulnar motor amplitudes from the second EMG, which were difficult to reconcile clinically when compared to findings from the first and third EMGs. Discrepancies in ulnar and median motor amplitudes between the 16- and 54-month EMGs were carefully reviewed by an independent, board-certified electromyographer. This variability was most likely attributable to technical factors such as electrode placement and possible volume conduction. While these inconsistencies introduced interpretive challenges, they did not alter the overall trajectory, which consistently demonstrated early reinnervation followed by sustained, though incomplete, recovery. Additionally, logistical challenges contributed to timing discrepancies between the second and third EMG assessments, mainly due to the patient’s significant geographical distance (4 h away) from our tertiary care center. Nevertheless, this detailed report substantially advances the understanding of peripheral nerve regeneration, providing unique insights into long-gap nerve allografting feasibility in young patients and delivering critical longitudinal electrodiagnostic data. Our findings underscore that nerve regeneration should be viewed as a dynamic and evolving process rather than a binary outcome, emphasizing the importance of sustained and multimodal evaluations. The combined use of strength assessments and sensory neurophysiological testing presented in this study further highlights the necessity of employing comprehensive evaluation protocols when analyzing complex nerve repairs.

Moving forward, studies incorporating larger cohorts, longer follow-up, and multimodal evaluation including EMG will be essential to define optimal techniques, guide patient selection, and ultimately improve outcomes in long-gap nerve reconstruction (Höke, 2006; Carvalho et al., 2019).

## 4 Conclusion

This case illustrates that meaningful functional recovery is achievable following reconstruction of a 68-mm ulnar nerve gap using processed nerve allograft, with the longest electrodiagnostic follow-up reported to date. Unlike prior cases involving delayed repair or adjunctive techniques, this patient underwent timely intervention and demonstrated early reinnervation with sustained motor unit recruitment over 4.5 years. Serial EMG was instrumental in tracking the course of regeneration and identifying late-stage conduction changes, reinforcing its role as a critical tool in postoperative surveillance. These findings support the clinical utility of processed allografts in managing large-gap peripheral nerve injuries and stresses the importance of objective, long-term follow-up in surgical outcome assessment. This study adds data to an area with limited longitudinal evidence and helps define the potential of allograft-based repair in complex large gap peripheral nerve reconstruction.

## Data Availability

The original contributions presented in this study are included in this article/Supplementary material, further inquiries can be directed to the corresponding author.
